# Staged complete revascularization or culprit-only percutaneous coronary intervention for multivessel coronary artery disease in patients with ST-segment elevation myocardial infarction and diabetes

**DOI:** 10.1186/s12933-019-0923-0

**Published:** 2019-09-17

**Authors:** Kongyong Cui, Shuzheng Lyu, Hong Liu, Xiantao Song, Fei Yuan, Feng Xu, Min Zhang, Wei Wang, Mingduo Zhang, Dongfeng Zhang, Jinfan Tian

**Affiliations:** Department of Cardiology, Beijing Anzhen Hospital, Capital Medical University and Beijing Institute of Heart, Lung and Blood Vessel Diseases, 2 Anzhen Road, Beijing, 100029 China

**Keywords:** Diabetes mellitus, Multivessel disease, Culprit-only percutaneous coronary artery intervention, Staged complete revascularization, Outcome

## Abstract

**Background:**

Recently, several randomized trials have noted improved outcomes with staged percutaneous coronary intervention (PCI) of nonculprit vessels in patients with ST-segment elevation myocardial infarction (STEMI) and multivessel disease. However, it remains unclear whether diabetes status affects the outcomes after different revascularization strategies. This study thus compared the impact of diabetes status on long-term outcomes after staged complete revascularization with that after culprit-only PCI.

**Methods:**

From January 2006 to December 2015, 371 diabetic patients (staged PCI: 164, culprit-only PCI: 207) and 834 nondiabetic patients (staged PCI: 412, culprit-only PCI: 422) with STEMI and multivessel disease were enrolled. The primary endpoint was 5-year major adverse cardiac and cerebrovascular event (MACCE), defined as a composite of all-cause death, myocardial infarction (MI), stroke or unplanned revascularization.

**Results:**

The rate of the 5-year composite primary endpoint for diabetic patients was close to that for nondiabetic patients (34.5% vs. 33.7%; adjusted hazard ratio [HR] 1.012, 95% confidence interval [CI] 0.815–1.255). In nondiabetic patients, the 5-year risks of MACCE (31.8% vs. 35.5%; adjusted HR 0.638, 95% CI 0.500–0.816), MI (4.6% vs. 9.2%; adjusted HR 0.358, 95% CI 0.200–0.641), unplanned revascularization (19.9% vs. 24.9%; adjusted HR 0.532, 95% CI 0.393–0.720), and the composite of cardiac death, MI or stroke (11.4% vs. 15.2%; adjusted HR 0.621, 95% CI 0.419–0.921) were significantly lower after staged PCI than after culprit-only PCI. In contrast, no significant difference was found between the two groups with respect to MACCE, MI, unplanned revascularization, and the composite of cardiac death, MI or stroke in diabetic patients. Significant interactions were found between diabetes status and revascularization assignment for the composite of cardiac death, MI or stroke (P_interaction_ = 0.013), MI (P_interaction_ = 0.005), and unplanned revascularization (P_interaction_ = 0.013) at 5 years. In addition, the interaction tended to be significant for the primary endpoint of MACCE (P_interaction_ = 0.053). Moreover, the results of propensity score-matching analysis were concordant with the overall analysis in both diabetic and nondiabetic population.

**Conclusions:**

In patients with STEMI and multivessel disease, diabetes is not an independent predictor of adverse cardiovascular events at 5 years. In nondiabetic patients, an approach of staged complete revascularization is superior to culprit-only PCI, whereas the advantage of staged PCI is attenuated in diabetic patients.

*Trial registration* This study was not registered in an open access database

## Background

Primary percutaneous coronary intervention (PCI) is currently the standard care for patients with ST-segment elevation myocardial infarction (STEMI). Approximately 50% of these patients have multivessel disease and present worse clinical outcomes compared with those having single-vessel disease [1, 2]. Although several previous small-scale randomized controlled trials (RCTs) and registries [3–8] supported a conservative approach for nonculprit diseases, recent landmark RCTs have improved outcomes with immediate or staged complete revascularization [9–12]. Accordingly, the latest European Society of Cardiology guideline upgraded the recommendation for nonculprit lesions revascularization during primary PCI or as a staged procedure over culprit-only PCI [13].

Diabetes is a strong independent predictor of adverse cardiovascular events in patients with coronary artery disease (CAD) [14–18]. Over recent decades, the prevalence of diabetes mellitus is dramatically increased from 108 million in 1980 to 451 million in 2017 [19, 20]. Generally, diabetic patients are prone to a diffuse and rapidly progressive form of atherosclerosis. This increases the risk of unfavorable clinical outcomes after revascularization [21, 22]. In this setting, diabetes might be an important consideration when choosing a revascularization strategy, i.e., staged complete revascularization or culprit-only PCI in patients with STEMI and multivessel disease.

Nevertheless, the relation between the effect of diabetes and different strategies remains underdetermined. These high-risk patients are generally underrepresented by RCTs, with a small proportion of diabetic patients enrolled [9–12]. In a study conducted by Hamza et al. [23], diabetic patients underwent complete revascularization with STEMI and multivessel disease were significantly associated with lower risk of adverse cardiovascular events than that in culprit-only PCI group. However, the limitations of their study were the small sample size and a short follow-up period of only 6 months. We therefore performed this study to compare the impact of diabetes status on long-term outcomes of patients with STEMI and multivessel disease after staged complete revascularization with that after culprit-only PCI.

## Methods

### Study design and population

The present report is a single-center, retrospective, observational study. The study design has been previously described [24]. Briefly, a total of 1205 patients with STEMI and multivessel disease who underwent primary PCI within 12 h from symptom onset underwent staged complete revascularization or culprit-only PCI between January 2006 and December 2015 in our center. The local ethical committee approved the study, and the written informed consent was waived because of the retrospective enrollment. In addition, patient records were anonymized and deidentified before database merging and analysis.

Diabetes mellitus was diagnosed based on previous medical records as well as therapeutic status of glucose-lowering therapy, i.e., insulin, oral hypoglycemic agents, diet and exercise. Multivessel disease was defined as the presence of ≥ 70% angiographic stenosis in ≥ 1 nonculprit major coronary arteries (with diameter ≥ 2.5 mm). Exclusion criteria were single-vessel disease (n = 1390), left main disease (n = 40), concomitant chronic total occlusion (n = 307), rescue PCI (n = 116), immediate complete revascularization (n = 81), undergoing coronary artery bypass graft surgery (n = 97), receiving medical therapy only (n = 34), or being dead during hospitalization (n = 16).

### Study procedures

All patients received loading doses of aspirin (300 mg), clopidogrel (600 mg) or ticagrelor (180 mg) before primary PCI. The culprit vessel was ascertained by evaluation of electrocardiographic changes, echocardiographic and angiographic findings. Primary PCI as well as the use of heparin, thrombus aspiration, and glycoprotein IIb/IIIa inhibitor were in compliance with the current guidelines and the operators’ routine practice [13, 25]. After the procedure, aspirin (100 mg/day) and clopidogrel (75 mg/day) or ticagrelor (180 mg/day) were prescribed at the same time every day. Culprit-only PCI was defined as the treatment of the culprit vessel only at the time of primary PCI without revascularization of nonculprit vessels during the following 30 days after primary PCI. In the staged PCI group, revascularization of significant nonculprit lesions was performed within 30 days after the procedure, which was determined by the physicians and/or patients. Contrast-induced acute kidney injury was defined as an increase in serum creatinine of ≥ 25% compared with baseline values or as an absolute increase in serum creatinine of ≥ 0.5 mg/dL (44.2 mmol/L) within 72 h after PCI [26, 27].

### Follow-up and endpoints

Demographics, cardiovascular risk factors, clinical characteristics, laboratory data, angiographic and procedural details were collected from hospital databases and recorded in a computerized database. Follow-up information was obtained from the review of hospital charts, clinical visits or telephone interviews, which were conducted by trained reviewers. In order to record at least 2-year follow-up information about all patients, we extended the follow-up period to May 31, 2018.

The primary endpoint was major adverse cardiac and cerebrovascular event (MACCE), defined as a composite of all-cause death, myocardial infarction (MI), stroke, or unplanned revascularization. Secondary outcomes included the individual components of the primary endpoint as well as cardiac death, and the composite of cardiac death, MI or stroke. All deaths were considered to be cardiac-related unless a non-cardiac origin was documented. Diagnosis of MI was made according to fourth universal definition of MI [28]. Stroke was defined as a new focal neurological deficit lasting > 24 h, which was confirmed by neurologists based on both clinical and radiographic criteria [29]. Unplanned revascularization was repeat PCI or coronary artery bypass grafting of any vessels excluding staged PCI. In addition, all the endpoints were verified and adjudicated by an independent clinical events committee (XTS, HL and SZL).

### Statistical analysis

Continuous variables were expressed as mean ± standard deviation or median (interquartile range), and were compared using the Student’s t test and Mann–Whitney U test according to different distributions. Categorical variables were expressed as number (percentage), and were compared using the Chi-square test or Fisher’s exact test. The Kaplan–Meier method was used to plot time-to-event curves, and differences were assessed using log-rank test. To find predictors of clinical events, Cox proportional hazard model analysis was conducted to provide adjusted hazard ratios (HRs) with 95% confidence intervals (CIs). Variables in Table 1 (without laboratory data) with P ≤ 0.1 at the univariate analysis were entered into multivariate Cox regression analysis. In particular, formal interaction testing was performed between diabetes status and revascularization treatment on all clinical outcomes.

**Table 1:** Baseline patient, angiographic and procedural characteristics according to diabetes status

Variable	No diabetes (n = 834)	Diabetes (n = 371)	P value
Age (years)	60 (51–68)	60 (53–68)	0.811
Male	675 (80.9)	280 (75.5)	0.031
Current smoker	467 (56.0)	183 (49.3)	0.032
Hypertension	495 (59.4)	242 (65.2)	0.053
Dyslipidemia	480 (57.6)	230 (62.0)	0.148
Previous myocardial infarction	39 (4.7)	26 (7.0)	0.098
Previous PCI	42 (5.0)	28 (7.5)	0.085
Previous stroke	74 (8.9)	44 (11.9)	0.107
Peripheral vascular disease	20 (2.4)	16 (4.3)	0.072
CKD in treatment	16 (1.9)	10 (2.7)	0.392
OSAHS	14 (1.7)	2 (0.5)	0.171
Heart rate (beats/min)	76 (68–85)	78 (70–85)	0.101
Systolic blood pressure (mmHg)	120 (108–130)	120 (110–130)	0.236
Laboratory data			
Peak troponin (μg/L)	68 (28–102)	73 (28–102)	0.808
Peak CK (U/L)	2101 (1124–3404)	1977 (987–3347)	0.204
Peak CK-MB (U/L)	227 (120–305)	173 (85–299)	< 0.001
Time from symptom onset to PCI (h)	5.0 (3.0–7.0)	5.0 (3.5–8.0)	0.009
Killip class III/IV	74 (8.9)	46 (12.4)	0.059
Radial artery access	295 (35.4)	151 (40.7)	0.077
No. narrowed coronary arteries			0.778
Two	580 (69.5)	255 (68.7)	
Three	254 (30.5)	116 (31.3)	
Culprit vessel			0.832
Left anterior descending	325 (39.0)	138 (37.2)	
Left circumflex	112 (13.4)	50 (13.5)	
Right	397 (47.6)	183 (49.3)	
Non-culprit artery			
Left anterior descending	370 (44.4)	178 (48.0)	0.245
Left circumflex	453 (54.3)	197 (53.1)	0.696
Right	266 (31.9)	111 (29.9)	0.495
Thrombus aspiration	582 (69.8)	234 (63.1)	0.021
No-reflow phenomenon	80 (9.6)	37 (10.0)	0.837
Intra-aortic balloon pump use	83 (10.0)	36 (9.7)	0.894
Glycoprotein IIb/IIIa inhibitor use	224 (26.9)	91 (24.5)	0.395
Temporary pacemaker	20 (2.4)	15 (4.0)	0.116
Defibrillator	43 (5.2)	18 (4.9)	0.824
Drug-eluting stent use	809 (97.0)	360 (97.0)	0.975
Type of stent			0.276
1st drug-eluting stent	634 (76.0)	265 (71.4)	
2nd drug-eluting stent	175 (21.0)	95 (25.6)	
Bare-mental stent	2 (0.2)	2 (0.5)	
PTCA	23 (2.8)	9 (2.4)	
Stent number	1 (1–2)	1 (1–2)	0.137
Total stent length (mm)	33 (24–48)	30 (24–42)	0.080
Minimum stent diameter (mm)	3.00 (2.50–3.50)	3.00 (2.50–3.50)	0.338
Medications at discharge			
Aspirin	833 (99.9)	371 (100.0)	1.000
P2Y12 receptor inhibitor	834 (100.0)	371 (100.0)	1.000
ACEI/ARB	617 (74.0)	269 (72.5)	0.592
β-blockers	693 (83.1)	331 (89.2)	0.006
Statins	826 (99.0)	370 (99.7)	0.289
Acute kidney injury^a^	177 (21.3)	74 (21.0)	0.602

*ACEI* angiotensin converting enzyme inhibitor, *ARB* angiotensin receptor blocker, *CKD* chronic kidney disease, *CK*-*MB* creatine kinase myocardial band, *OSAHS* obstructive sleep apnea-hypopnea syndrome, *PCI* percutaneous coronary intervention, *PTCA* percutaneous transluminal coronary angioplasty

^a^Data of acute kidney injury was obtained from 1200 (99.6%) patients

To adjust for potential confounders from the real world, a double 1:1 propensity score-matching analysis (staged PCI vs. culprit-only PCI groups) without replacement, on the basis of the nearest neighbor in terms of Mahalanobis distance with a caliper of 0.02, was performed in each subgroup of patients, i.e., nondiabetic and diabetic patients. To estimate the propensity score, a logistic regression model was used including variables of age, gender, current smoking, hypertension, previous MI, previous PCI, peripheral vascular disease, chronic kidney disease, time from symptom onset to PCI, heart rate, access site of PCI, Killip class III/IV, number of diseased vessels, culprit vessel of left anterior descending coronary artery, nonculprit vessel of left anterior descending coronary artery, thrombus aspiration, intra-aortic balloon pump, stent length, use of angiotensin-converting enzyme inhibitors or angiotensin receptor blockers and use of β-blockers. In addition, to assess the robustness of the results, long-term outcomes of patients undergoing staged PCI within 10 days were compared with those in culprit-only PCI group not undergoing revascularization of nonculprit vessels during the following 10 days after primary PCI in both nondiabetic and diabetic population.

Statistical analyses were conducted using SPSS 23.0 (SPSS Inc., Chicago, Illinois, USA) and STATA 12.0 (StataCorp, College Station, Texas, USA). A two-sided P value of < 0.05 was considered to indicate statistical significance.

## Results

### Baseline patient, angiographic and procedural characteristics

Among the 1205 patients with STEMI and multivessel disease who received staged complete revascularization (n = 576) or culprit-only PCI (n = 629), 371 (30.8%) had diabetes mellitus, of which 164 (44.2%) received staged PCI and 207 (55.8%) underwent culprit-only PCI. Among the remaining 834 nondiabetic patients, 412 (49.4%) received staged PCI and 422 (50.6%) underwent culprit-only PCI. Staged PCI was performed after a median time of 6 days in both nondiabetic and diabetic cohorts. The staged procedures were performed within 10 days after primary PCI in 89.0% (n = 146) of the diabetic patients and 90.3% (n = 372) of the nondiabetic patients. The mean follow-up period was 5.01 years.

Compared with nondiabetic patients, diabetic patients were less likely to be male (P = 0.031) and current smokers (P = 0.032) and to receive thrombus aspiration (P = 0.021), but were more likely to receive β-blockers (P = 0.006) with longer time from symptom to intervention (P = 0.009) (Table 1). In diabetic patients, those who underwent staged PCI were more likely to be male (P = 0.006), had less culprit vessels of left anterior descending coronary artery (P = 0.003), and were less frequently to receive transradial PCI (P < 0.001) and defibrillator (P = 0.016) than those who received culprit-only PCI. In nondiabetic patients, those who underwent staged PCI were younger (P = 0.001), had lower prevalence rates of chronic kidney disease (P = 0.013) and Killip class III/IV (P = 0.010), had more three-vessel disease (P < 0.001), nonculprit vessels of left anterior descending coronary artery (P = 0.003), were more frequently to use intra-aortic balloon pump (P = 0.006), angiotensin converting enzyme inhibitor/angiotensin receptor blocker (P < 0.001) during hospitalization, and less likely to receive transradial PCI (P < 0.001) and temporary pacemaker (P = 0.027). Besides, patients who underwent staged PCI had lower heart rates (P = 0.010), shorter time from symptom to intervention (P = 0.004), and shorter total stent length (P = 0.010) than those who underwent culprit-only PCI (Table 2).

**Table 2:** Baseline patient, angiographic and procedural characteristics according to diabetes status and revascularization assignment

Variable	No diabetes (n = 834)			Diabetes (n = 371)		
	Culprit-only PCI (n = 422)	Staged PCI (n = 412)	P value	Culprit-only PCI (n = 207)	Staged PCI (n = 164)	P value
Age (years)	61 (52–70)	58 (50–66)	0.001	61 (53–69)	59 (51–66)	0.055
Male	334 (79.1)	341 (82.8)	0.183	145 (70.0)	135 (82.3)	0.006
Current smoker	225 (53.3)	242 (58.7)	0.115	98 (47.3)	85 (51.8)	0.391
Hypertension	262 (62.1)	233 (56.6)	0.104	140 (67.6)	102 (62.2)	0.275
Dyslipidemia	241 (57.1)	239 (58.0)	0.792	123 (59.4)	107 (65.2)	0.251
Previous myocardial infarction	23 (5.5)	16 (3.9)	0.284	16 (7.7)	10 (6.1)	0.541
Previous PCI	22 (5.2)	20 (4.9)	0.813	16 (7.7)	12 (7.3)	0.881
Previous stroke	43 (10.2)	31 (7.5)	0.176	28 (13.5)	16 (9.8)	0.265
Peripheral vascular disease	8 (1.9)	12 (2.9)	0.337	9 (4.3)	7 (4.3)	0.970
CKD in treatment	13 (3.1)	3 (0.7)	0.013	6 (2.9)	4 (2.4)	1.000
OSAHS	9 (2.1)	5 (1.2)	0.302	2 (1.0)	0 (0)	0.505
Heart rate (beats/min)	76 (69–86)	75 (67–84)	0.010	78 (70–86)	78 (70–85)	0.884
Systolic blood pressure (mmHg)	120 (107–130)	120 (109–130)	0.151	120 (108–130)	120 (110–132)	0.619
Laboratory data						
Peak troponin (μg/L)	68 (25–101)	68 (30–114)	0.076	76 (27–102)	69 (28–108)	0.735
Peak CK (U/L)	2110 (1088–3391)	2076 (1156–3474)	0.611	1974 (982–3360)	1986 (1012–3311)	0.918
Peck CK-MB (U/L)	239 (111–304)	217 (128–307)	0.865	180 (89–293)	168 (68–300)	0.963
Time from symptom onset to PCI (h)	5.0 (3.0–8.0)	4.0 (3.0–7.0)	0.004	5.0 (3.0–8.0)	5.0 (3.5–8.0)	0.701
Killip class III/IV	48 (11.4)	26 (6.3)	0.010	31 (15.0)	15 (9.1)	0.091
Radial artery access	192 (45.5)	103 (25.0)	<* 0.001*	101 (48.8)	50 (30.5)	< 0.001
No. narrowed coronary arteries			*< 0.001*			0.082
Two	317 (75.1)	263 (63.8)		150 (72.5)	105 (64.0)	
Three	105 (24.9)	149 (36.2)		57 (27.5)	59 (36.0)	
Culprit vessel			0.094			0.003
Left anterior descending	168 (39.8)	157 (38.1)		88 (42.5)	50 (30.5)	
Left circumflex	46 (10.9)	66 (16.0)		18 (8.7)	32 (19.5)	
Right	208 (49.3)	189 (45.9)		101 (48.8)	82 (50.0)	
Non-culprit artery						
Left anterior descending	166 (39.3)	204 (49.5)	0.003	90 (43.5)	88 (53.7)	0.051
Left circumflex	246 (58.3)	207 (50.2)	0.020	115 (55.6)	82 (50.0)	0.287
Right	115 (27.3)	151 (36.7)	0.004	58 (28.0)	53 (32.3)	0.369
Thrombus aspiration	284 (67.3)	298 (72.3)	0.114	133 (64.3)	101 (61.6)	0.597
No-reflow phenomenon	46 (10.9)	34 (8.3)	0.194	23 (11.1)	14 (8.5)	0.411
Intra-aortic balloon pump use	30 (7.1)	53 (12.9)	0.006	21 (10.1)	15 (9.1)	0.747
Glycoprotein IIb/IIIa inhibitor use	103 (24.4)	121 (29.4)	0.106	55 (26.6)	36 (22.0)	0.304
Temporary pacemaker	15 (3.6)	5 (1.2)	0.027	12 (5.8)	3 (1.8)	0.054
Defibrillator	24 (5.7)	19 (4.6)	0.483	15 (7.2)	3 (1.8)	0.016
Drug-eluting stent use	405 (96.0)	404 (98.1)	0.077	199 (96.1)	161 (98.2)	0.359
Type of stent			0.280			0.153
1st drug-eluting stent	314 (74.4)	320 (77.7)		140 (67.6)	125 (76.2)	
2nd drug-eluting stent	91 (21.6)	84 (20.4)		59 (28.5)	36 (22.0)	
Bare-mental stent	1 (0.2)	1 (0.2)		2 (1.0)	0 (0)	
PTCA	16 (3.8)	7 (1.7)		6 (2.9)	3 (1.8)	
Stent number	1 (1–2)	1 (1–2)	0.213	1 (1–2)	1 (1–2)	0.269
Total stent length (mm)	33 (24–51)	31 (24–46)	0.010	33 (24–44)	29 (23–41)	0.141
Minimum stent diameter (mm)	3.0 (2.5–3.5)	3.0 (2.5–3.5)	0.672	3.0 (2.5–3.5)	3.0 (2.5–3.5)	0.561
Medications at discharge						
Aspirin	421 (99.8)	412 (100)	1.000	207 (100)	164 (100)	1.000
P2Y12 receptor inhibitor	422 (100)	412 (100)	1.000	207 (100)	164 (100)	1.000
ACEI/ARB	290 (68.7)	327 (79.4)	< 0.001	143 (69.1)	126 (76.8)	0.097
β-blockers	359 (85.1)	334 (81.1)	0.123	185 (89.4)	146 (89.0)	0.915
Statins	418 (99.1)	408 (99.0)	1.000	207 (100)	163 (99.4)	0.442
Acute kidney injury^a^	80 (19.1)	97 (23.6)	0.113	37 (18.0)	37 (22.6)	0.272

*ACEI* angiotensin converting enzyme inhibitor, *ARB* angiotensin receptor blocker, *CKD* chronic kidney disease, *CK*-*MB* creatine kinase myocardial band, *OSAHS* obstructive sleep apnea-hypopnea syndrome, *PCI* percutaneous coronary intervention, *PTCA* percutaneous transluminal coronary angioplasty

^a^Data of acute kidney injury was obtained from 1200 (99.6%) patients

### Comparison of 5-year outcomes between diabetic and nondiabetic patients

**Fig. 1:**
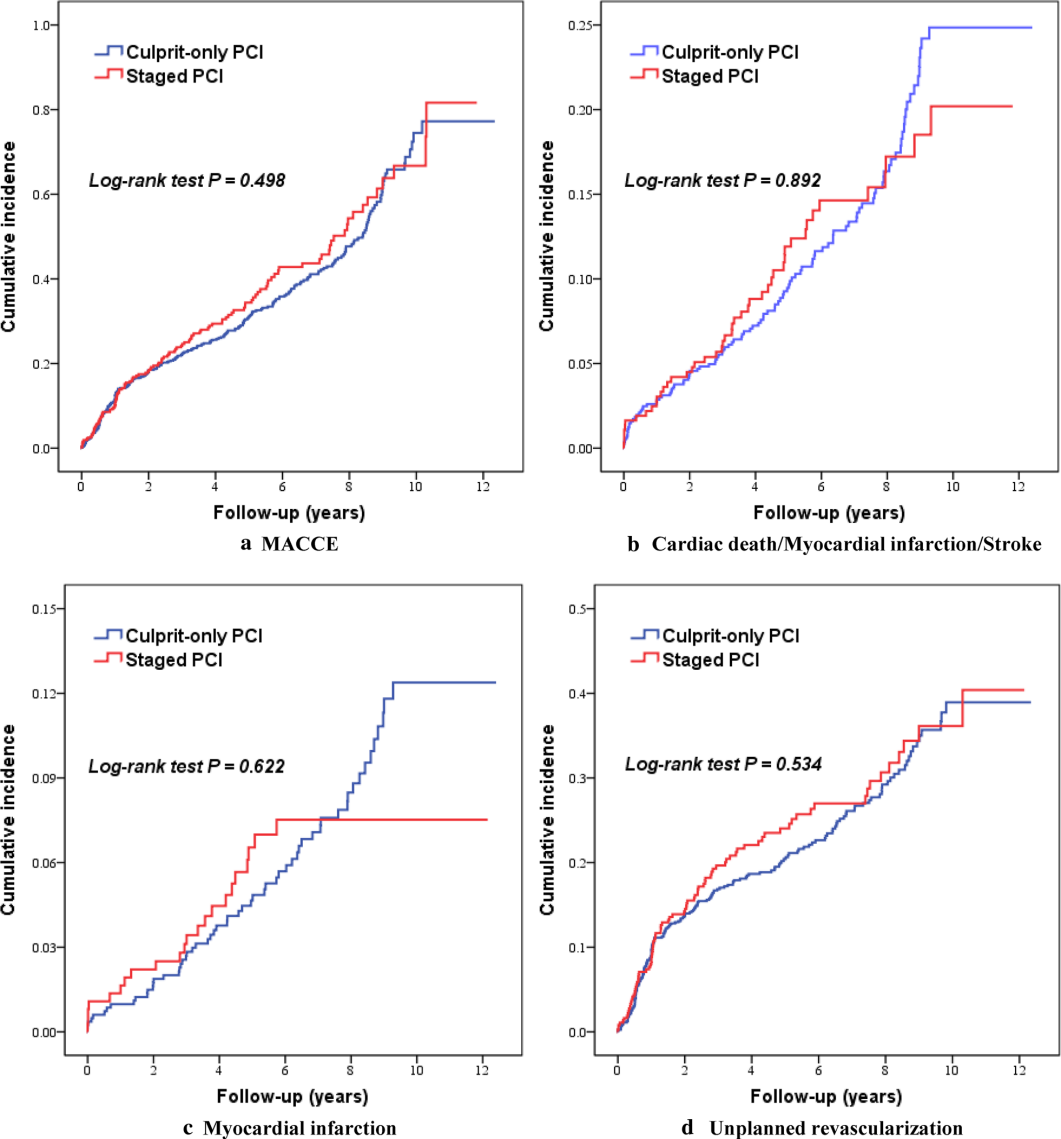
Kaplan–Meier curves of clinical outcomes in patients with versus without diabetes mellitus. *MACCE* major adverse cardiac and cerebrovascular event, *PCI* percutaneous coronary intervention

Clinical outcomes according to diabetes status are shown in Table 3 and Fig. 1. The 5-year incidences of MACCE (34.5% vs. 33.7%; HR 1.075, 95% CI 0.872 to 1.325) and a composite of cardiac death, MI or stroke (12.4% vs. 13.3%; HR 0.976, 95% CI 0.692 to 1.377) were similar in diabetic patients and nondiabetic patients. In addition, the risks of all-cause death, cardiac death, MI, stroke, and unplanned revascularization were not significantly different between those two groups.

**Table 3:** The comparison of 5-year outcomes between the diabetic group and nondiabetic group

Clinical endpoint	Overall (n = 1205)		Crude HR (95% CI)	Adjusted HR (95% CI)
	No diabetes (n = 834)	Diabetes (n = 371)		
MACCE	281 (33.7)	128 (34.5)	1.075 (0.872–1.325)	1.012 (0.815–1.255)
Cardiac death/MI/stroke	111 (13.3)	46 (12.4)	0.976 (0.692–1.377)	0.869 (0.608–1.242)
All-cause death	80 (9.6)	32 (8.6)	0.935 (0.620–1.409)	0.785 (0.512–1.203)
Cardiac death	37 (4.4)	19 (5.1)	1.210 (0.696–2.104)	0.982 (0.549–1.757)
MI	58 (7.0)	22 (5.9)	0.884 (0.541–1.444)	0.855 (0.515–1.419)
Stroke	20 (2.4)	11 (3.0)	1.280 (0.613–2.671)	1.148 (0.540–2.440)
Unplanned revascularization	187 (22.4)	88 (23.7)	1.084 (0.841–1.396)	1.065 (0.826–1.374)

*CI* confidence interval, *HR* hazard ratio, *MACCE* major adverse cardiac and cerebrovascular event, *MI* myocardial infarction

After adjusting potential confounders, diabetes was not independently associated with the primary endpoint of MACCE (Adjusted HR 1.012, 95% CI 0.815 to 1.255), and the composite of cardiac death, MI or stroke (Adjusted HR 0.869, 95% CI 0.608 to 1.242) at 5 years. Notably, the strategy of culprit-only PCI (P = 0.010), previous stroke (P = 0.012), chronic kidney disease (P = 0.016), and high systolic blood pressure (P = 0.021) were independently associated with the higher incidence of MACCE at 5 years (Table 4).

**Table 4:** Cox proportional hazards analysis of predictors of the primary endpoint at 5 years

Variable	Univariate analysis		Multivariate analysis	
	HR (95% CI)	P value	HR (95% CI)	P value
Diabetes (vs. no diabetes)	1.075 (0.872–1.325)	0.498	1.012 (0.815–1.255)	0.916
Staged PCI (vs. culprit-only PCI)	0.721 (0.593–0.878)	0.001	0.766 (0.626–0.937)	0.010
Male (vs. female)	0.791 (0.630–0.995)	0.045	0.892 (0.703–1.131)	0.346
Previous stroke	1.593 (1.191–2.131)	0.002	1.475 (1.088–2.000)	0.012
Peripheral vascular disease	1.584 (0.974–2.574)	0.064	1.563 (0.953–2.561)	0.077
Chronic kidney disease	2.379 (1.419–3.991)	0.001	1.922 (1.131–3.268)	0.016
Heart rate	1.007 (1.001–1.013)	0.028	1.005 (0.998–1.011)	0.140
Systolic blood pressure	1.005 (1.000–1.010)	0.040	1.006 (1.001–1.011)	0.021
Killip class III/IV	1.361 (1.010–1.834)	0.043	1.291 (0.934–1.785)	0.122
Stent number	1.150 (0.993–1.331)	0.062	1.081 (0.797–1.465)	0.618
Total stent length	1.007 (1.001–1.013)	0.022	1.004 (0.992–1.015)	0.509
No-reflow phenomenon	1.336 (0.991–1.801)	0.057	1.248 (0.917–1.701)	0.159
Use of aspirin	0.069 (0.010–0.498)	0.008	0.176 (0.023–1.349)	0.095

*CI* confidence interval, *HR* hazard ratio, *PCI* percutaneous coronary intervention

### Comparison of 5-year outcomes between staged complete revascularization and culprit-only PCI

**Fig. 2:**
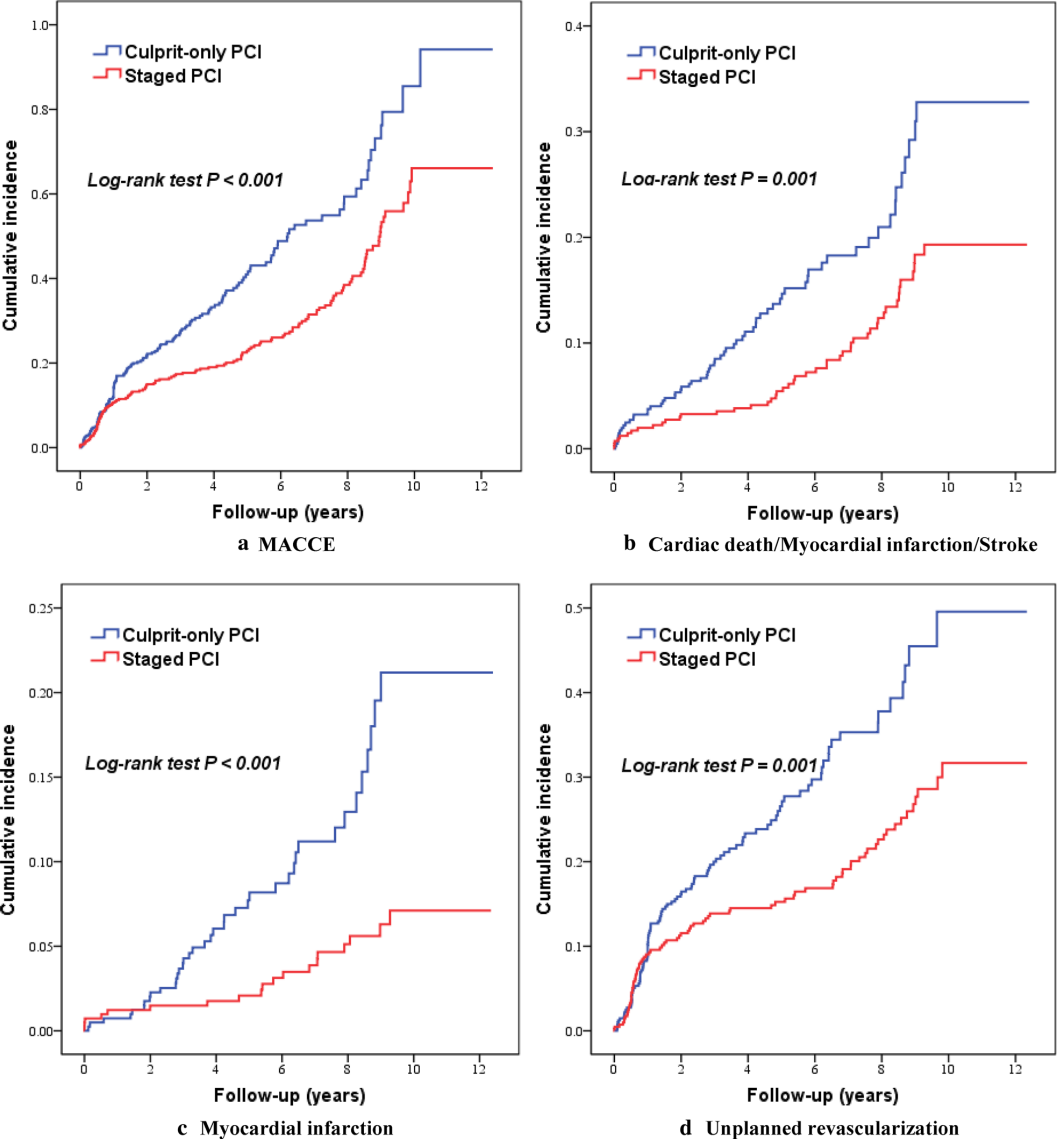
Kaplan–Meier curves of clinical outcomes in nondiabetic patients. *MACCE* major adverse cardiac and cerebrovascular event, *PCI* percutaneous coronary intervention

Among nondiabetic patients, patients who received staged PCI presented lower risks of MACCE (31.8% vs. 35.5%; HR 0.643, 95% CI 0.507 to 0.815), MI (4.6% vs. 9.2%; HR 0.346, 95% CI 0.199 to 0.601), unplanned revascularization (19.9% vs. 24.9%; HR 0.625, 95% CI 0.466 to 0.837), and the composite of cardiac death, MI or stroke (11.4% vs. 15.2%; HR 0.529, 95% CI 0.362 to 0.774) than those who underwent culprit-only PCI (Table 5 and Fig. 2). No significant difference was found between the two revascularization strategies with respect to all-cause mortality, cardiac mortality, and stroke. After the potential confounders were adjusted, staged PCI was associated with a decrease in the risk of the primary endpoint of MACCE (Adjusted HR 0.638, 95% CI 0.500 to 0.816), MI (Adjusted HR 0.358, 95% CI 0.200 to 0.641), unplanned revascularization (Adjusted HR 0.532, 95% CI 0.393 to 0.720), and the composite of cardiac death, MI or stroke (Adjusted HR 0.621, 95% CI 0.419 to 0.921) in nondiabetic patients.

**Table 5:** Five-year outcomes according to diabetes status and revascularization assignment

Clinical endpoint	No diabetes (n = 834)		Crude HR (95% CI)	Adjusted HR (95% CI)	Diabetes (n = 371)		Crude HR (95% CI)	Adjusted HR (95% CI)	P for interaction
	Culprit-only PCI (n = 422)	Staged PCI (n = 412)			Culprit-only PCI (n = 207)	Staged PCI (n = 164)			
MACCE	150 (35.5)	131 (31.8)	0.643(0.507–0.815)	0.638 (0.500–0.816)	66 (31.9)	62 (37.8)	0.939 (0.662–1.331)	0.986 (0.683–1.422)	*0.053*
Cardiac death/MI/stroke	64 (15.2)	47 (11.4)	0.529 (0.362–0.774)	0.621 (0.419–0.921)	23 (11.1)	23 (14.0)	1.042 (0.583–1.862)	1.593 (0.846–3.000)	*0.013*
All-cause death	39 (9.2)	41 (10.0)	0.808 (0.520–1.257)	1.281 (0.803–2.042)	18 (8.7)	14 (8.5)	0.709 (0.350–0.434)	0.876 (0.404–1.898)	0.410
Cardiac death	17 (4.0)	20 (4.9)	0.913 (0.476–1.753)	1.650 (0.817–3.335)	11 (5.3)	8 (4.9)	0.685 (0.274–1.717)	0.840 (0.304–2.316)	0.284
MI	39 (9.2)	19 (4.6)	0.346 (0.199–0.601)	0.358(0.200–0.641)	10 (4.8)	12 (7.3)	1.339 (0.578–3.103)	1.599 (0.663–3.858)	*0.005*
Stroke	12 (2.8)	8 (1.9)	0.517 (0.210–1.272)	0.574 (0.231–1.426)	6 (2.9)	5 (3.0)	0.878 (0.267–2.885)	0.857 (0.248–2.964)	0.610
Unplanned revascularization	105 (24.9)	82 (19.9)	0.625 (0.466–0.837)	0.532 (0.393–0.720)	45 (21.7)	43 (26.2)	1.045 (0.687–1.590)	1.038 (0.672–1.605)	*0.013*

*CI* confidence interval, *HR* hazard ratio, *MACCE* major adverse cardiovascular and cerebrovascular event, *MI* myocardial infarction, *PCI* percutaneous coronary intervention

**Fig. 3:**
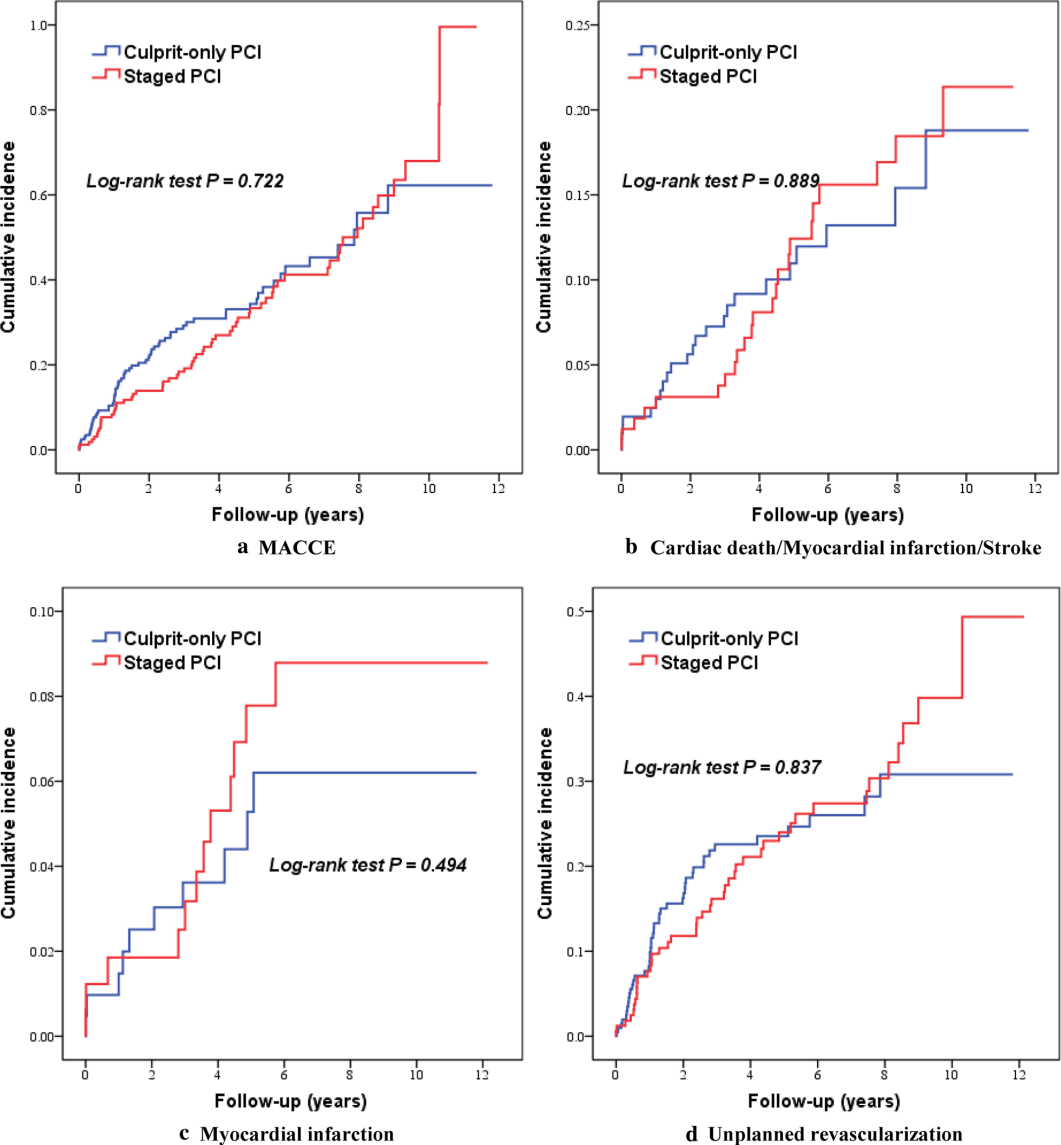
Kaplan–Meier curves of clinical outcomes in diabetic patients. *MACCE* major adverse cardiac and cerebrovascular event, *PCI* percutaneous coronary intervention

In diabetic patients, the incidences of the primary endpoint of MACCE (37.8% vs. 31.9%; HR 0.939, 95% CI 0.662 to 1.331) and the secondary outcomes were comparable between the two revascularization therapies (Table 5 and Fig. 3). After the potential confounders were adjusted, staged PCI was not independently associated with MACCE (Adjusted HR 0.986, 95% CI 0.683 to 1.422) and the secondary endpoints of the composite of cardiac death, MI or stroke (Adjusted HR 1.593, 95% CI 0.846 to 3.000), all-cause death (Adjusted HR 0.876, 95% CI 0.404 to 1.898), cardiac death (Adjusted HR 0.840, 95% CI 0.304 to 2.316), MI (Adjusted HR 1.599, 95% CI 0.663 to 3.858), stroke (Adjusted HR 0.857, 95% CI 0.248 to 2.964) and unplanned revascularization (Adjusted HR 1.038, 95% CI 0.672 to 1.605) at 5 years.

By formal interaction testing, significant interactions were found between diabetes status and revascularization assignment for the composite of cardiac death, MI or stroke (P_interaction_ = 0.013), MI (P_interaction_ = 0.005), and unplanned revascularization (P_interaction_ = 0.013) at 5 years. In addition, the interaction tended to be significant for the primary endpoint of MACCE (P_interaction_ = 0.053). However, there were no significant interactions between diabetes status and treatment for all-cause mortality, cardiac mortality and stroke (Table 5).

### Propensity score-matching analysis

After propensity score-matching, 127 matched pairs for diabetic patients with STEMI and multivessel disease and 280 matched pairs for nondiabetic patients with STEMI and multivessel disease were generated. All the matched variables were well balanced for both diabetic and nondiabetic cohorts, with postmatching absolute standardized differences < 10% (Additional file 1: Figure S1). No significant differences for main baseline patient, angiographic and procedural characteristics were present between the staged PCI and culprit-only PCI groups in both diabetic and nondiabetic cohorts (Additional file 1: Table S1). Diabetic patients undergoing staged PCI were less likely to receive defibrillator compared with those in culprit-only group (P = 0.010). In nondiabetic population, compared with those in culprit-only group, patients in the staged PCI group were less likely to receive temporary pacemaker (P = 0.020) and associated with higher risk of acute kidney injury after PCI (P = 0.049).

Among nondiabetic patients, patients undergoing staged PCI were associated with lower risks of MACCE (HR 0.583, 95% CI 0.432 to 0.787), MI (HR 0.275, 95% CI 0.134 to 0.563), unplanned revascularization (HR 0.546, 95% CI 0.380 to 0.783), and the composite of cardiac death, MI or stroke (HR 0.498, 95% CI 0.311 to 0.798) compared with those undergoing culprit-only PCI (Additional file 1: Table S2 and Figure S2A). In diabetic patients, the risks of the primary endpoint of MACCE (HR 1.271, 95% CI 0.820 to 1.971) and all the secondary outcomes were comparable between the two strategies (Additional file 1: Table S2 and Figure S2B). Furthermore, there were significant interactions between diabetes status and revascularization assignment for MACCE (P_interaction_ = 0.004), MI (P_interaction_ = 0.004), unplanned revascularization (P_interaction_ = 0.005), and the composite of cardiac death, MI or stroke (P_interaction_ = 0.007).

### Sensitivity analysis

Sensitivity analysis comparing patients undergoing staged complete revascularization within 10 days after primary PCI versus those undergoing culprit-only PCI was concordant with the overall analysis in both diabetic and nondiabetic population. However, diabetic patients undergoing staged PCI was associated with higher risk of MI than those undergoing culprit-only PCI (Adjusted HR 2.617, 95% CI 1.057 to 6.481) (Additional file 1: Tables S3, S4 and Figure S3).

## Discussion

During the 10-year study, diabetes was present in 30.8% of the patients with STEMI and multivessel disease who underwent primary PCI in our center. Multivariate analysis showed that diabetes mellitus was not independently associated with the primary endpoint of MACCE or the secondary outcomes at 5 years. Compared with culprit-only PCI, staged complete revascularization was associated with lower risks of MACCE, MI, unplanned revascularization and the composite of cardiac death, MI or stroke in nondiabetic patients. However, no significant difference was found between the two revascularization strategies in terms of all the outcomes in diabetic patients. Besides, significant interactions between diabetes status and treatment for MI, unplanned revascularization and the composite of cardiac death, MI or stroke at 5 years were found. Furthermore, these findings were demonstrated by propensity score-matching analysis.

Patients with STEMI and multivessel disease were associated with worse outcomes than those with single-vessel disease [1, 2]. However, the management of nonculprit lesions has been fiercely debated for two decades until the recent publication of the landmark RCTs [9–12]. The Preventative Angioplasty in Myocardial Infarction trial showed that preventive PCI of nonculprit lesions significantly reduced the risk of a composite endpoint of cardiac death, MI, and refractory angina at 23 months [9]. The Complete Versus culprit-Lesion only PRimary PCI trial indicated that patients who received in-hospital complete revascularization had lower composite risk of all-cause death, recurrent MI, heart failure, and ischemia-driven revascularization at 1 year [10]. In addition, the Third DANish Study of Optimal Acute Treatment of Patients with STEMI-PRImary PCI in MULTIvessel Disease and the Compare-Acute trials indicated significant benefit of immediate or complete revascularization regarding adverse cardiac events compared with culprit-only PCI [11, 12]. Furthermore, the studies conducted by Cui et al. and Toyota et al. with 5-year information confirmed and extended the results of previous studies with short- or medium-term follow-up period [24, 30].

Diabetes mellitus is both an important risk factor for the development of CAD [20, 31] and a major determinant of poor clinical outcomes in patients with CAD [14–18]. Patients with diabetes mellitus often have a high incidence of complex disease with smaller vessel size, longer lesion length, and higher plaque burden [32]. The Improving Care for Cardiovascular Disease in China-Acute Coronary Syndrome Project which included 63,450 patients from 150 tertiary hospitals revealed that the prevalence of diabetes/possible diabetes was 36.8% in STEMI patients, which was a little higher than the finding of our study. In addition, diabetic/possible diabetic patients had 2.4-fold increased risk of in-hospital mortality and a twofold increased risk of a combination of cardiac death, recurrent MI, stent thrombosis or stroke compared with nondiabetic patients [14]. Jung et al. [15] reported that people with diabetes had a two- to sixfold higher risk of major adverse cardiac events than people without diabetes in South Korea. A report from Spain showed that patients with MI and diabetes had a significantly 15% higher in-hospital mortality than nondiabetic patients [16]. A systematic review and meta-analysis with a total of 1,225,174 patients revealed an increased risk of early mortality (odds ratio 1.66, 95% CI 1.59 to 1.74) and 6–12-month mortality (odds ratio 1.86, 95% CI 1.75 to 1.97) in diabetic patients with acute coronary syndrome [17]. Besides, Klempfner et al. [18] enrolled 11, 472 patients with acute coronary syndrome found that diabetes was independently associated with a significantly increased mortality risk (39%) at 1 year compared with nondiabetic patients. Moreover, the incidence of ischemic events was consistently higher in diabetic patients after PCI or coronary artery bypass graft surgery [21, 22]. The latest guideline has classified STEMI patients with diabetes as a special population and presented specific sections for the management of these patients in consideration of their extremely high risk [13]. Therefore, diabetes status might be a major factor in the choice of revascularization strategy in patients with STEMI and multivessel disease.

Unfortunately, it remains undetermined whether diabetes has an effect on the outcomes of these patients who received staged complete revascularization or culprit-only PCI. Only a small number of patients with diabetes were included in previous RCTs and this high-risk group of patients were underrepresented [9–12]. Hamza et al. enrolled 100 diabetic patients with STMEI and multivessel disease to randomly receive staged complete revascularization (n = 50) or culprit-only PCI (n = 50). After 6-month follow-up, they found that staged complete revascularization was significantly associated with a reduction in major adverse cardiac events (6% vs. 24%, P = 0.01), primarily due to reduction in ischemia-driven revascularization in the complete PCI group (2% vs. 12%; P = 0.047). However, their sample size was relatively small and the follow-up period was relatively short [23]. Therefore, it is necessary to determine whether the effect of diabetes on clinical outcomes differs according to different revascularization strategies.

In this study, STEMI patients with and without diabetes mellitus showed similar risks of ischemic events. Furthermore, diabetes was not a predictor of the primary endpoint of MACCE or the secondary outcomes at 5 years in multivariate analysis, while the strategy of culprit-only PCI was an independent predictor of the less favorable outcomes in these patients. Patients with STEMI and multivessel disease was a higher-risk population in STEMI patients, thus the impact of revascularization strategy on prognosis is more important than the impact of diabetes status on prognosis in our study. Although with increased risk of perioperative events, early revascularization of nonculprit lesions can reduce ischemic burden, stabilize vulnerable plaque, and reduce the long-term incidence of ischemic events [33]. Nonetheless, the comparable results between the diabetic and nondiabetic groups here could be partially explained, since data on the length of illness and details of antidiabetic therapy was not available in our study, which might have an effect on prognosis in diabetic patients. Actually, inappropriate antidiabetic therapy can significantly increase the risk of mortality [34].

The most important finding of the present study might be that the interactions between diabetes status and revascularization assignment tended to be significant for the outcomes of MACCE, MI, unplanned revascularization, and the composite of cardiac death, MI or stroke at 5 years, which were confirmed by propensity score-matching analysis. In nondiabetic patients, the 5-year risks of MACCE, MI, unplanned revascularization, and the composite of cardiac death, MI or stroke were significantly lower in staged PCI group than those in culprit-only PCI group, whereas the incidences of all the outcomes were similar between the two revascularization strategies in diabetic patients. In other words, the strategy of staged complete revascularization lost its advantage in patients with diabetes and multivessel disease, which was contrary to the results of study conducted by Hamza et al. In clinical scenarios, the diffuse and rapidly progressive forms of CAD in diabetic patients may lead to more stent implantation characterized by longer length and smaller diameter, which is associated with worse outcomes. Although the new-generation drug-eluting stent has been widely used in clinical practice, the morbidity and mortality are still high in diabetic patients undergoing PCI and diabetes mellitus remains a risk factor for restenosis and stent thrombosis [32, 35]. Considering the staged PCI of nonculprit vessels brings no additional benefits to diabetic patients with multivessel disease as compared with culprit-only PCI, it becomes even more important to choose an optimal hypoglycemic regimen in this population. Recently, several studies have found that the new antidiabetic drugs, i.e., sodium-glucose cotransporter 2 inhibitors and glucagon-like peptide 1 agonists can lower blood glucose levels and mortality risks [36–39]. Henceforward, these new types of drugs should be given a full consideration in the treatment of diabetic patients with STEMI and multivessel disease.

### Limitations

There are several limitations of our study. First, as a single-center, nonrandomized study, our research is limited by unbalanced baseline characteristics and selection bias. Although we performed rigorous multivariable-adjusted analysis and propensity score-matching analysis, there might still be some unmeasured confounders. Second, our results were mainly derived from subgroup analysis of a cohort study, thus we might have inadequate statistical power to detect differences in clinical events in diabetic patients and the results should be interpreted as hypothesis generating. Moreover, the number of subjects with diabetes was modest (371), and possibly not all confounders were identified. Therefore, further larger-scale investigation in dedicated trials of diabetic patients is warranted. Third, the data on length of illness and details of antidiabetic therapy were not collected in the study. Finally, the significance of nonculprit lesions was routinely assessed on angiography other than ischemia testing, for example, fractional flow reserve or noninvasive physiological stress test for most patients.

## Conclusions

In patients with STEMI and multivessel disease, diabetes mellitus is not an independent predictor of adverse cardiovascular events at 5 years. In nondiabetic patients, an approach of staged complete revascularization is superior to culprit-only PCI, whereas the advantage of staged PCI is attenuated in diabetic patients. Further studies are needed to evaluate the prognostic impact of diabetes on outcomes in patients with STEMI and multivessel disease requiring revascularization procedures.

## Supplementary information

**Additional file 1. Table S1.** Baseline patient, angiographic and procedural characteristics according to diabetes status and revascularization assignment in propensity-matched population. **Table S2.** Five-year outcomes according to diabetes status and revascularization assignment in propensity-matched population. **Table S3.** Baseline patient, angiographic and procedural characteristics according to diabetes status and revascularization assignment in sensitivity analysis. **Table S4.** Five-year outcomes according to diabetes status and revascularization assignment in sensitivity analysis. **Figure S1.** Absolute standard difference before and after propensity score-matching in (A) nondiabetic population and (B) diabetic population. **Figure S2.** Kaplan–Meier curves of clinical outcomes for (A) nondiabetic patients and (B) diabetic patients in propensity-matched population. **Figure S3.** Kaplan–Meier curves of clinical outcomes for (A) nondiabetic patients and (B) diabetic patients in sensitivity analysis.

## Data Availability

The datasets used and/or analyzed during the current study are available from the corresponding author on reasonable request.

## Abbreviations

PCI: percutaneous coronary intervention
STEMI: ST-segment elevation myocardial infarction
RCT: randomized controlled trial
CAD: coronary artery disease
MACCE: major adverse cardiac and cerebrovascular event
MI: myocardial infarction
HR: hazard ratio
CI: confidence interval
