# Supplementary material for: Staged complete revascularization or culprit-only percutaneous coronary intervention for multivessel coronary artery disease in patients with ST-segment elevation myocardial infarction and diabetes
Source: Cardiovasc Diabetol. 2019 Sep 17;18:119. doi: 10.1186/s12933-019-0923-0 (PMC6749697; doi:10.1186/s12933-019-0923-0)
Supplement: Supplementary file 1 — Additional file 1. Table S1. Baseline patient, angiographic and procedural characteristics according to diabetes status and revascularization assignment in propensity-matched population. Table S2. Five-year outcomes according to diabetes status and revascularization assignment in propensity-matched population. Table S3. Baseline patient, angiographic and procedural characteristics according to diabetes status and revascularization assignment in sensitivity analysis. Table S4. Five-year outcomes according to diabetes status and revascularization assignment in sensitivity analysis. Figure S1. Absolute standard difference before and after propensity score-matching in (A) nondiabetic population and (B) diabetic population. Figure S2. Kaplan–Meier curves of clinical outcomes for (A) nondiabetic patients and (B) diabetic patients in propensity-matched population. Figure S3. Kaplan–Meier curves of clinical outcomes for (A) nondiabetic patients and (B) diabetic patients in sensitivity analysis. [file 12933_2019_923_MOESM1_ESM.docx]

**Table S1** Baseline patient, angiographic and procedural characteristics according to diabetes status and revascularization assignment in propensity-matched population.

**Table S2** Five-year outcomes according to diabetes status and revascularization assignment in propensity-matched population.

**Table S3** Baseline patient, angiographic and procedural characteristics according to diabetes status and revascularization assignment in sensitivity analysis.

**Table S4** Five-year outcomes according to diabetes status and revascularization assignment in sensitivity analysis.

**Figure S1** Absolute standard difference before and after propensity score-matching in **(A)** nondiabetic population and **(B)** diabetic population.

**Figure S2** Kaplan-Meier curves of clinical outcomes for **(A)** nondiabetic patients and **(B)** diabetic patients in propensity-matched population.

**Figure S3** Kaplan-Meier curves of clinical outcomes for **(A)** nondiabetic patients and **(B)** diabetic patients in sensitivity analysis.

**Table S1** Baseline patient, angiographic and procedural characteristics according to diabetes status and revascularization assignment in propensity-matched population.

| Variable | No diabetes (n=560) | | | Diabetes (n=254) | | |
| --- | --- | --- | --- | --- | --- | --- |
|  | Culprit-only PCI (n=280) | Staged PCI (n=280) | P value | Culprit-only PCI (n=127) | Staged PCI (n=127) | P value |
| Age (years) | 60 (51-68) | 60 (52-68) | 0.755 | 59 (52-66) | 60 (51-65) | 0.959 |
| Male | 231 (82.5) | 226 (80.7) | 0.586 | 98 (77.2) | 99 (78.0) | 0.880 |
| Current smoker | 158 (56.4) | 159 (56.8) | 0.932 | 64 (50.4) | 62 (48.8) | 0.802 |
| Hypertension | 167 (59.6) | 177 (63.2) | 0.385 | 81 (63.8) | 81 (63.8) | 1.000 |
| Dyslipidemia | 154 (55.0) | 152 (54.3) | 0.865 | 74 (58.3) | 80 (63.0) | 0.441 |
| Previous myocardial infarction | 12 (4.3) | 9 (3.2) | 0.505 | 8 (6.3) | 9 (7.1) | 0.802 |
| Previous PCI | 14 (5.0) | 9 (3.2) | 0.287 | 11 (8.7) | 10 (7.9) | 0.820 |
| Previous stroke | 27 (9.6) | 21 (7.5) | 0.365 | 20 (15.7) | 13 (10.2) | 0.191 |
| Peripheral vascular disease | 6 (2.1) | 6 (2.1) | 1.000 | 5 (3.9) | 6 (4.7) | 0.758 |
| CKD in treatment | 2 (0.7) | 2 (0.7) | 1.000 | 3 (2.4) | 4 (3.1) | 1.000 |
| OSAHS | 6 (2.1) | 5 (1.8) | 0.761 | 1 (0.8) | 0 (0) | 1.000 |
| Heart rate (beats/min) | 76 (69-85) | 75 (67-85) | 0.391 | 76 (70-85) | 77 (70-84) | 0.813 |
| Systolic blood pressure (mmHg) | 120 (109-130) | 120 (110-130) | 0.351 | 120 (110-130) | 120 (110-140) | 0.484 |
| Laboratory data |  |  |  |  |  |  |
| Peak troponin (μg/L) | 70 (25-102) | 68 (28-102) | 0.447 | 61 (26-102) | 67 (27-107) | 0.684 |
| Peak CK (U/L) | 2000 (1003-3382) | 2118 (1145-3414) | 0.356 | 1600 (888-2718) | 1977 (1021-3255) | 0.125 |
| Peck CK-MB (U/L) | 231 (100-305) | 209 (130-302) | 0.829 | 154 (78-253) | 166 (75-300) | 0.166 |
| Time from symptom onset to PCI (hours) | 5.0 (3.0-7.5) | 5.0 (3.0-7.0) | 0.612 | 5.0 (6.5-8.0) | 6.0 (4.0-9.0) | 0.394 |
| Killip class III/IV | 22 (7.9) | 22 (7.9) | 1.000 | 11 (8.7) | 14 (11.0) | 0.527 |
| Radial artery access | 96 (34.3) | 99 (35.4) | 0.790 | 48 (37.8) | 47 (37.0) | 0.897 |
| Three-vessel disease | 84 (30.0) | 76 (27.1) | 0.454 | 44 (34.6) | 40 (31.5) | 0.594 |
| Culprit vessel of LAD | 114 (40.7) | 123 (43.9) | 0.441 | 41 (32.3) | 44 (34.6) | 0.690 |
| Non-culprit artery of LAD | 116 (41.4) | 110 (39.3) | 0.605 | 65 (51.2) | 62 (48.8) | 0.707 |
| Thrombus aspiration | 188 (67.1) | 188 (67.1) | 1.000 | 80 (63.0) | 80 (63.0) | 1.000 |
| No-reflow phenomenon | 36 (12.9) | 23 (8.2) | 0.074 | 16 (12.6) | 11 (8.7) | 0.309 |
| Intra-aortic balloon pump use | 26 (9.3) | 21 (7.5) | 0.446 | 13 (10.2) | 12 (9.4) | 0.833 |
| Glycoprotein IIb/IIIa inhibitor use | 68 (24.3) | 69 (24.6) | 0.922 | 34 (26.8) | 28 (22.0) | 0.381 |
| Temporary pacemaker | 10 (3.6) | 2 (0.7) | **0.020** | 7 (5.5) | 3 (2.4) | 0.197 |
| Defibrillator | 11 (3.9) | 10 (3.6) | 0.824 | 9 (7.1) | 1 (0.8) | **0.010** |
| Drug-eluting stent use | 279 (99.6) | 279 (99.6) | 1.000 | 125 (98.4) | 127 (100.0) | 0.498 |
| Type of stent |  |  | 1.000 |  |  | 0.116 |
| 1st drug-eluting stent | 217 (77.5) | 217 (77.5) |  | 89 (70.1) | 99 (78.0) |  |
| 2nd drug-eluting stent | 62 (22.1) | 62 (22.1) |  | 36 (28.3) | 28 (22.0) |  |
| Bare-mental stent | 1 (0.4) | 1 (0.4) |  | 2 (1.6) | 0 (0) |  |
| Stent number | 1 (1-2) | 1 (1-2) | 0.992 | 1 (1-2) | 1 (1-2) | 0.766 |
| Total stent length (mm) | 33 (24-48) | 33 (24-47) | 0.634 | 30 (24-42) | 30 (23-46) | 0.987 |
| Minimum stent diameter (mm) | 3.0 (2.5-3.5) | 3.0 (2.5-3.5) | 0.825 | 3.0 (2.5-3.5) | 3.0 (2.5-3.0) | 0.132 |
| Medications at discharge |  | | | | | |
| Aspirin | 279 (99.6) | 280 (100.0) | 1.000 | 127 (100.0) | 127 (100.0) | 1.000 |
| P2Y12 receptor inhibitor | 280 (100.0) | 280 (100.0) | 1.000 | 127 (100.0) | 127 (100.0) | 1.000 |
| ACEI/ARB | 204 (72.9) | 211 (75.4) | 0.499 | 97 (76.4) | 94 (74.0) | 0.663 |
| β-blockers | 233 (83.2) | 234 (83.6) | 0.910 | 114 (89.8) | 114 (89.8) | 1.000 |
| Statins | 278 (99.3) | 279 (99.6) | 1.000 | 127 (100.0) | 126 (99.2) | 1.000 |
| Acute kidney injury***** | 48 (17.3) | 67 (24.0) | **0.049** | 16 (12.7) | 27 (21.3) | 0.070 |

*ACEI* angiotensin converting enzyme inhibitor, *ARB* angiotensin receptor blocker, *CKD* chronic kidney disease, *CK-MB* creatine kinase myocardial band, *LAD* left anterior descending coronary artery, *OSAHS* obstructive sleep apnea-hypopnea syndrome, *PCI* percutaneous coronary intervention.

* Data of acute kidney injury was obtained from 812 (99.8%) patients.

**Table S2** Five-year outcomes according to diabetes status and revascularization assignment in propensity-matched population.

| Clinical endpoint | No diabetes (n=560) | | HR  (95% CI) | Diabetes (n=254) | | HR  (95% CI) | P for interaction |
| --- | --- | --- | --- | --- | --- | --- | --- |
|  | Culprit-only PCI (n=280) | Staged PCI (n=280) |  | Culprit-only PCI (n=127) | Staged PCI (n=127) |  |  |
| MACCE | 102 (36.4) | 75 (26.8) | **0.583 (0.432-0.787)** | 34 (26.8) | 49 (38.6) | 1.271 (0.820-1.971) | **0.004** |
| Cardiac death/MI/stroke | 46 (16.4) | 28 (10.0) | **0.498 (0.311-0.798)** | 10 (7.9) | 19 (15.0) | 1.723 (0.801-3.709) | **0.007** |
| All-cause death | 21 (7.5) | 22 (7.9) | 0.910 (0.500-1.655) | 9 (7.1) | 11 (8.7) | 1.010 (0.418-2.440) | 0.848 |
| Cardiac death | 9 (3.2) | 14 (5.0) | 1.367 (0.591-3.160) | 4 (3.1) | 6 (4.7) | 1.288 (0.363-4.574) | 0.939 |
| MI | 30 (10.7) | 10 (3.6) | **0.275 (0.134-0.563)** | 5 (3.9) | 10 (7.9) | 1.861 (0.636-5.446) | **0.004** |
| Stroke | 8 (2.9) | 4 (1.4) | 0.431 (0.130-1.433) | 5 (3.9) | 3 (2.4) | 0.529 (0.126-2.214) | 0.830 |
| Unplanned revascularization | 75 (26.8) | 49 (17.5) | **0.546 (0.380-0.783)** | 23 (18.1) | 34 (26.8) | 1.374 (0.809-2.333) | **0.005** |

*CI* confidence interval, *HR* hazard ratio, *MACCE* major adverse cardiovascular and cerebrovascular event, *MI* myocardial infarction, *PCI* percutaneous coronary intervention.

**Table S3** Baseline patient, angiographic and procedural characteristics according to diabetes status and revascularization assignment in sensitivity analysis.

| Variable | No diabetes (n=834) | | | Diabetes (n=371) | | |
| --- | --- | --- | --- | --- | --- | --- |
|  | Culprit-only PCI (n=462) | Staged PCI (n=372) | P value | Culprit-only PCI (n=225) | Staged PCI (n=146) | P value |
| Age (years) | 61 (52-70) | 58 (50-66) | **< 0.001** | 61 (54-69) | 59 (50-65) | **0.018** |
| Male | 368 (79.7) | 307 (82.5) | 0.294 | 157 (69.8) | 123 (84.2) | **0.002** |
| Current smoker | 216 (46.8) | 151 (40.6) | 0.075 | 121 (53.8) | 67 (45.9) | 0.138 |
| Hypertension | 285 (61.7) | 210 (56.5) | 0.126 | 150 (66.7) | 92 (63.0) | 0.470 |
| Dyslipidemia | 261 (56.5) | 219 (58.9) | 0.490 | 132 (58.7) | 98 (67.1) | 0.101 |
| Previous myocardial infarction | 23 (5.0) | 16 (4.3) | 0.645 | 16 (7.1) | 10 (6.8) | 0.923 |
| Previous PCI | 22 (4.8) | 20 (5.4) | 0.687 | 16 (7.1) | 21 (8.2) | 0.693 |
| Previous stroke | 46 (10.0) | 28 (7.5) | 0.220 | 31 (13.8) | 13 (8.9) | 0.156 |
| Peripheral vascular disease | 8 (1.7) | 12 (3.2) | 0.161 | 9 (4.0) | 7 (4.8) | 0.713 |
| CKD in treatment | 15 (3.2) | 1 (0.3) | **0.002** | 7 (3.1) | 3 (2.1) | 0.746 |
| OSAHS | 10 (2.2) | 4 (1.1) | 0.224 | 2 (0.9) | 0 (0) | 0.521 |
| Heart rate (beats/min) | 77 (69-86) | 75 (67-84) | **0.004** | 78 (70-86) | 79 (70-85) | 0.828 |
| Systolic blood pressure (mmHg) | 120 (107-130) | 120 (110-130) | 0.331 | 120 (108-130) | 120 (110-140) | 0.276 |
| Laboratory data |  |  |  |  |  |  |
| Peak troponin (μg/L) | 70 (26-101) | 67 (28-115) | 0.237 | 79 (27-102) | 63 (28-107) | 0.719 |
| Peak CK (U/L) | 2124 (1088-3404) | 2058 (1160-3414) | 0.996 | 2089 (1030-3451) | 1835 (894-3189) | 0.236 |
| Peck CK-MB (U/L) | 239 (112-304) | 215 (128-307) | 0.950 | 185 (94-300) | 162 (65-295) | 0.339 |
| Time from symptom onset to PCI (hours) | 5.0 (3.0-8.0) | 4.0 (3.0-6.9) | **0.004** | 5.0 (3.5-8.0) | 5.0 (3.5-9.0) | 0.752 |
| Killip class III/IV | 56 (12.1) | 18 (4.8) | **< 0.001** | 35 (15.6) | 11 (7.5) | **0.022** |
| Radial artery access | 207 (44.8) | 88 (23.7) | **< 0.001** | 106 (47.1) | 45 (30.8) | **0.002** |
| No. narrowed coronary arteries |  |  | **0.005** |  |  | 0.220 |
| Two | 340 (73.6) | 240 (64.5) |  | 160 (71.1) | 95 (65.1) |  |
| Three | 122 (26.4) | 132 (35.5) |  | 65 (28.9) | 51 (34.9) |  |
| Culprit vessel |  |  | 0.113 |  |  | **0.010** |
| Left anterior descending | 182 (39.4) | 143 (38.4) |  | 94 (41.8) | 44 (30.1) |  |
| Left circumflex | 52 (11.3) | 60 (16.1) |  | 22 (9.8) | 28 (19.2) |  |
| Right | 228 (49.4) | 169 (45.4) |  | 109 (48.4) | 74 (50.7) |  |
| Non-culprit artery |  | | | | | |
| Left anterior descending | 187 (40.5) | 183 (49.2) | **0.012** | 101 (44.9) | 77 (52.7) | 0.139 |
| Left circumflex | 266 (57.6) | 187 (50.3) | **0.035** | 123 (54.7) | 74 (50.7) | 0.453 |
| Right | 131 (28.4) | 135 (36.3) | **0.015** | 65 (28.9) | 46 (31.5) | 0.591 |
| Thrombus aspiration | 312 (67.5) | 270 (72.6) | 0.115 | 145 (64.4) | 89 (61.0) | 0.497 |
| No-reflow phenomenon | 51 (11.0) | 29 (7.8) | 0.114 | 27 (12.0) | 10 (6.8) | 0.106 |
| Intra-aortic balloon pump use | 37 (8.0) | 46 (12.4) | **0.037** | 22 (9.8) | 14 (9.6) | 0.952 |
| Glycoprotein IIb/IIIa inhibitor use | 115 (24.9) | 109 (29.3) | 0.153 | 58 (25.8) | 33 (22.6) | 0.487 |
| Temporary pacemaker | 18 (3.9) | 2 (0.5) | **0.002** | 13 (5.8) | 2 (1.4) | **0.035** |
| Defibrillator | 26 (5.6) | 17 (4.6) | 0.492 | 17 (7.6) | 1 (0.7) | **0.003** |
| Drug-eluting stent use | 445 (96.3) | 364 (97.8) | 0.198 | 217 (96.4) | 143 (97.9) | 0.538 |
| Type of stent |  |  | 0.489 |  |  | 0.178 |
| 1st drug-eluting stent | 345 (74.7) | 289 (77.7) |  | 153 (68.0) | 112 (76.7) |  |
| 2nd drug-eluting stent | 100 (21.6) | 75 (20.2) |  | 64 (28.4) | 31 (21.2) |  |
| Bare-mental stent | 1 (0.2) | 1 (0.3) |  | 2 (0.9) | 0 (0) |  |
| PTCA | 16 (3.5) | 7 (1.9) |  | 6 (2.7) | 3 (2.1) |  |
| Stent number | 1 (1-2) | 1 (1-2) | 0.222 | 1 (1-2) | 1 (1-2) | 0.137 |
| Total stent length (mm) | 33 (24-51) | 30 (24-45) | **0.004** | 33 (24-46) | 29 (23-41) | 0.112 |
| Minimum stent diameter (mm) | 3.0 (2.5-3.5) | 3.0 (2.5-3.5) | 0.750 | 3.0 (2.5-3.5) | 3.0 (2.5-3.5) | 0.991 |
| Medications at discharge |  | | | | | |
| Aspirin | 461 (99.8) | 372 (100.0) | 1.000 | 225 (100.0) | 146 (100.0) | 1.000 |
| P2Y12 receptor inhibitor | 462 (100.0) | 372 (100.0) | 1.000 | 225 (100.0) | 146 (100.0) | 1.000 |
| ACEI/ARB | 321 (69.5) | 296 (79.6) | **0.001** | 158 (70.2) | 111 (76.0) | 0.221 |
| β-blockers | 393 (85.1) | 300 (80.6) | 0.090 | 201 (89.3) | 130 (89.0) | 0.929 |
| Statins | 458 (99.1) | 368 (98.9) | 1.000 | 225 (100.0) | 145 (99.3) | 0.394 |
| Acute kidney injury***** | 91 (19.8) | 86 (23.2) | 0.241 | 42 (18.8) | 32 (21.9) | 0.457 |

*ACEI* angiotensin converting enzyme inhibitor, *ARB* angiotensin receptor blocker, *CKD* chronic kidney disease, *CK-MB* creatine kinase myocardial band, *OSAHS* obstructive sleep apnea-hypopnea syndrome, *PCI* percutaneous coronary intervention, *PTCA* percutaneous transluminal coronary angioplasty.

* Data of acute kidney injury was obtained from 1200 (99.6%) patients.

**Table S4** Five-year outcomes according to diabetes status and revascularization assignment in sensitivity analysis.

| Clinical endpoint | No diabetes (n=834) | | Crude HR  (95% CI) | Adjusted HR  (95% CI) | Diabetes (n=371) | | Crude HR  (95% CI) | Adjusted HR  (95% CI) | P for interaction |
| --- | --- | --- | --- | --- | --- | --- | --- | --- | --- |
|  | Culprit-only PCI (n=462) | Staged PCI (n=372) |  |  | Culprit-only PCI (n=225) | Staged PCI (n=146) |  |  |  |
| MACCE | 163 (35.3) | 118 (31.7) | **0.655 (0.515-0.833)** | **0.675 (0.525-0.866)** | 74 (32.9) | 54 (37.0) | 0.913 (0.641-1.299) | 1.035 (0.711-1.507) | **0.063** |
| Cardiac death/MI/stroke | 71 (15.4) | 40 (10.8) | **0.492 (0.333-0.728)** | **0.581 (0.386-0.875)** | 26 (11.6) | 20 (13.7) | 0.993 (0.553-1.783) | 1.665 (0.878-3.156) | **0.006** |
| All-cause death | 49 (10.6) | 31 (8.3) | **0.578 (0.367-0.909)** | 0.974 (0.595-1.595) | 22 (9.8) | 10 (6.8) | 0.513 (0.242-1.089) | 0.849 (0.383-1.880) | 0.774 |
| Cardiac death | 22 (4.8) | 15 (4.0) | 0.636 (0.328-1.233) | 1.286 (0.610-2.711) | 13 (5.8) | 6 (4.1) | 0.539 (0.204-1.426) | 1.025 (0.499-2.104) | 0.668 |
| MI | 40 (8.7) | 18 (4.8) | **0.395 (0.226-0.693)** | **0.403 (0.223-0.730)** | 10 (4.4) | 12 (8.2) | 1.695 (0.732-3.925) | **2.617 (1.057-6.481)** | **0.001** |
| Stroke | 13 (2.8) | 7 (1.9) | 0.509 (0.202-1.283) | 0.586 (0.227-1.510) | 7 (3.1) | 4 (2.7) | 0.760 (0.222-2.601) | 0.690 (0.187-2.550) | 0.843 |
| Unplanned revascularization | 108 (23.4) | 79 (21.2) | **0.734 (0.547-0.984)** | **0.644 (0.477-0.870)** | 48 (21.3) | 40 (27.4) | 1.161 (0.763-1.768) | 1.149 (0.739-1.789) | **0.034** |

*CI* confidence interval, *HR* hazard ratio, *MACCE* major adverse cardiovascular and cerebrovascular event, *MI* myocardial infarction, *PCI* percutaneous coronary intervention.

**Figure S1** Absolute standard difference before and after propensity score-matching in (A) nondiabetic population and (B) diabetic population.

*ACEI* angiotensin converting enzyme inhibitor, *ARB* angiotensin receptor blocker, *LAD* left anterior descending coronary artery, *PCI* percutaneous coronary intervention.


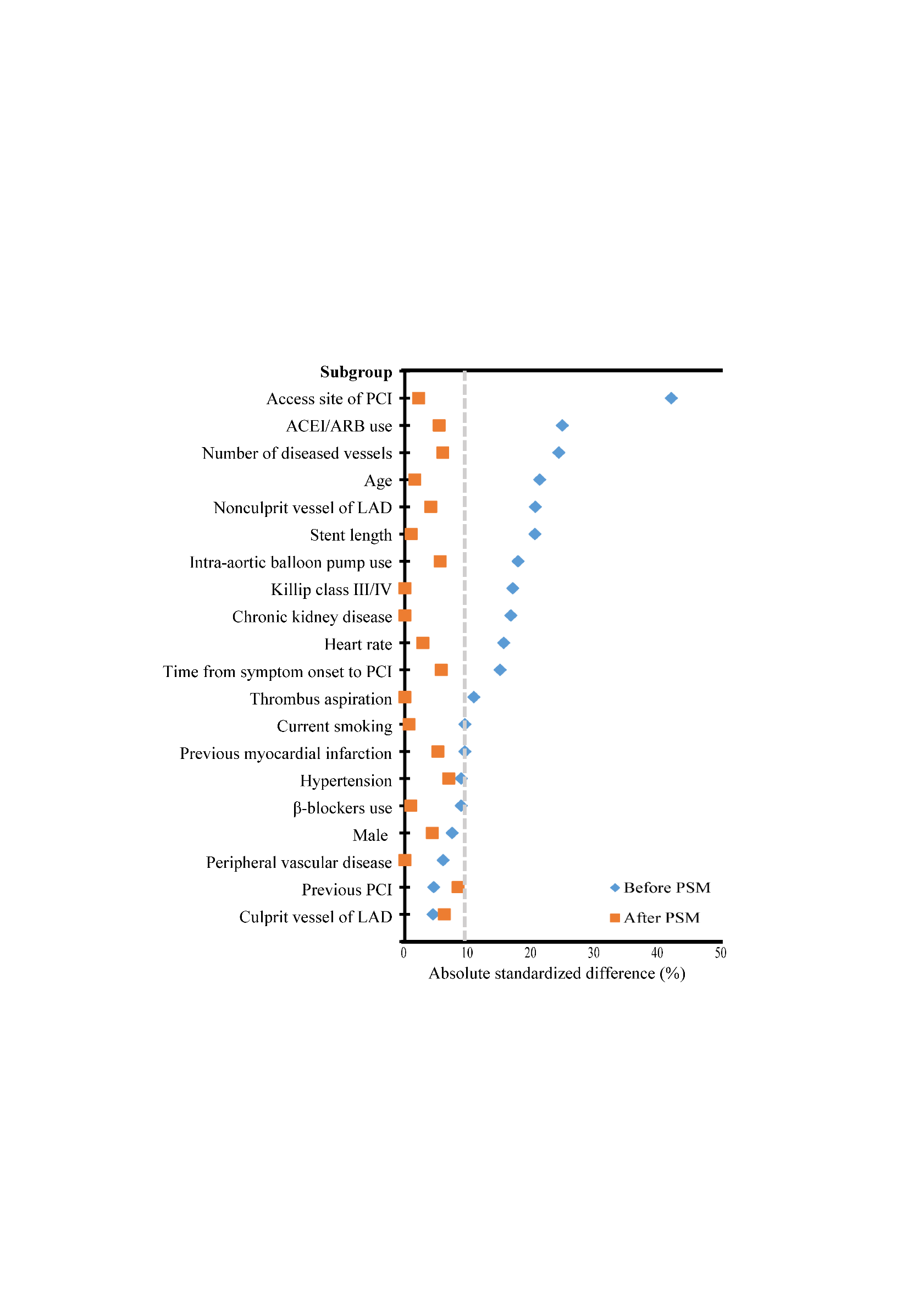
**(A)**

**Figure S1** Absolute standard difference before and after propensity score-matching in **(A)** nondiabetic population and **(B)** diabetic population.

*ACEI* angiotensin converting enzyme inhibitor, *ARB* angiotensin receptor blocker, *LAD* left anterior descending coronary artery, *PCI* percutaneous coronary intervention.

**
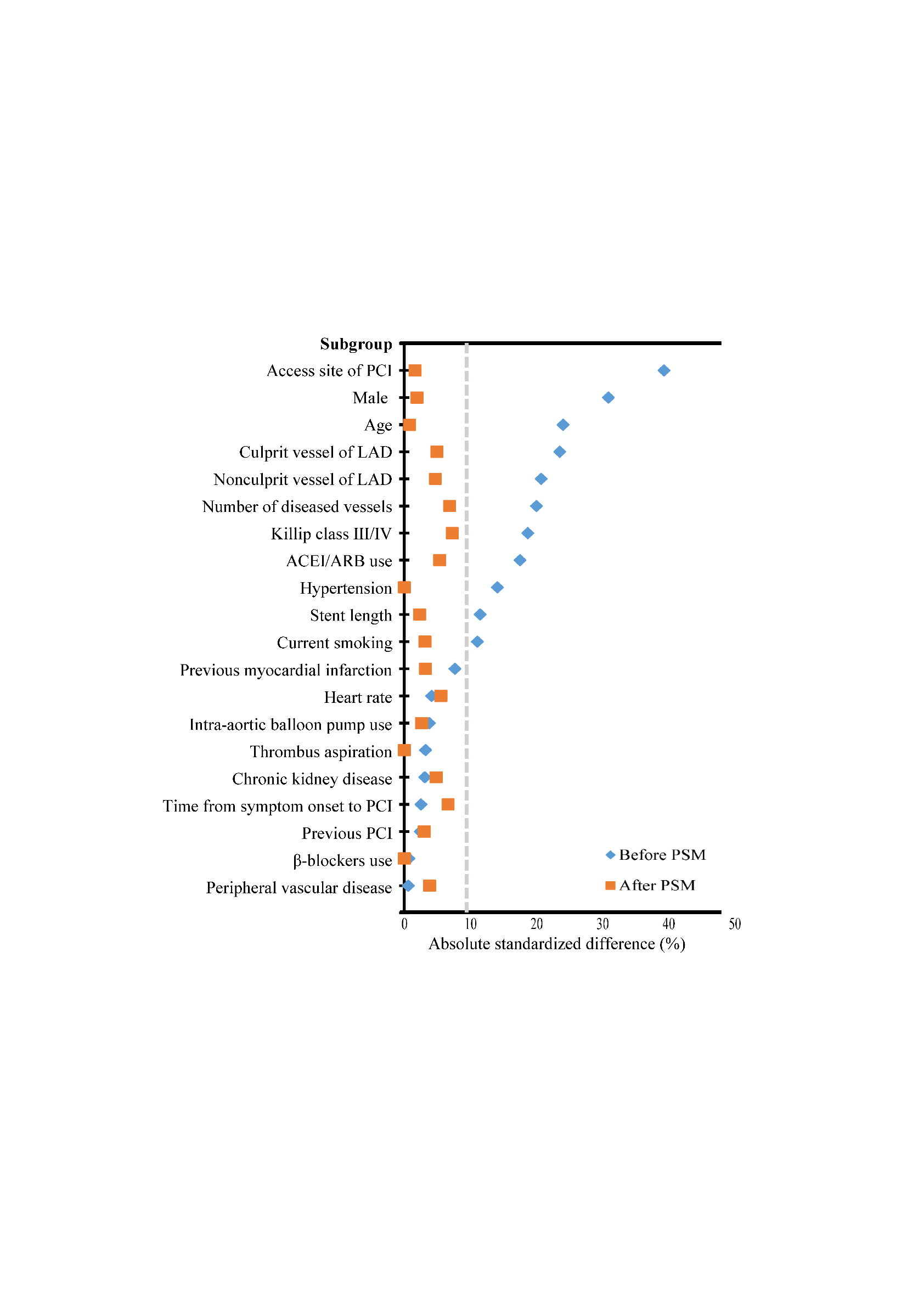
(B)**

**Figure S2** Kaplan-Meier curves of clinical outcomes for **(A)** nondiabetic patients and **(B)** diabetic patients in propensity-matched population.

*MACCE* major adverse cardiac and cerebrovascular event, *PCI* percutaneous coronary intervention.

**
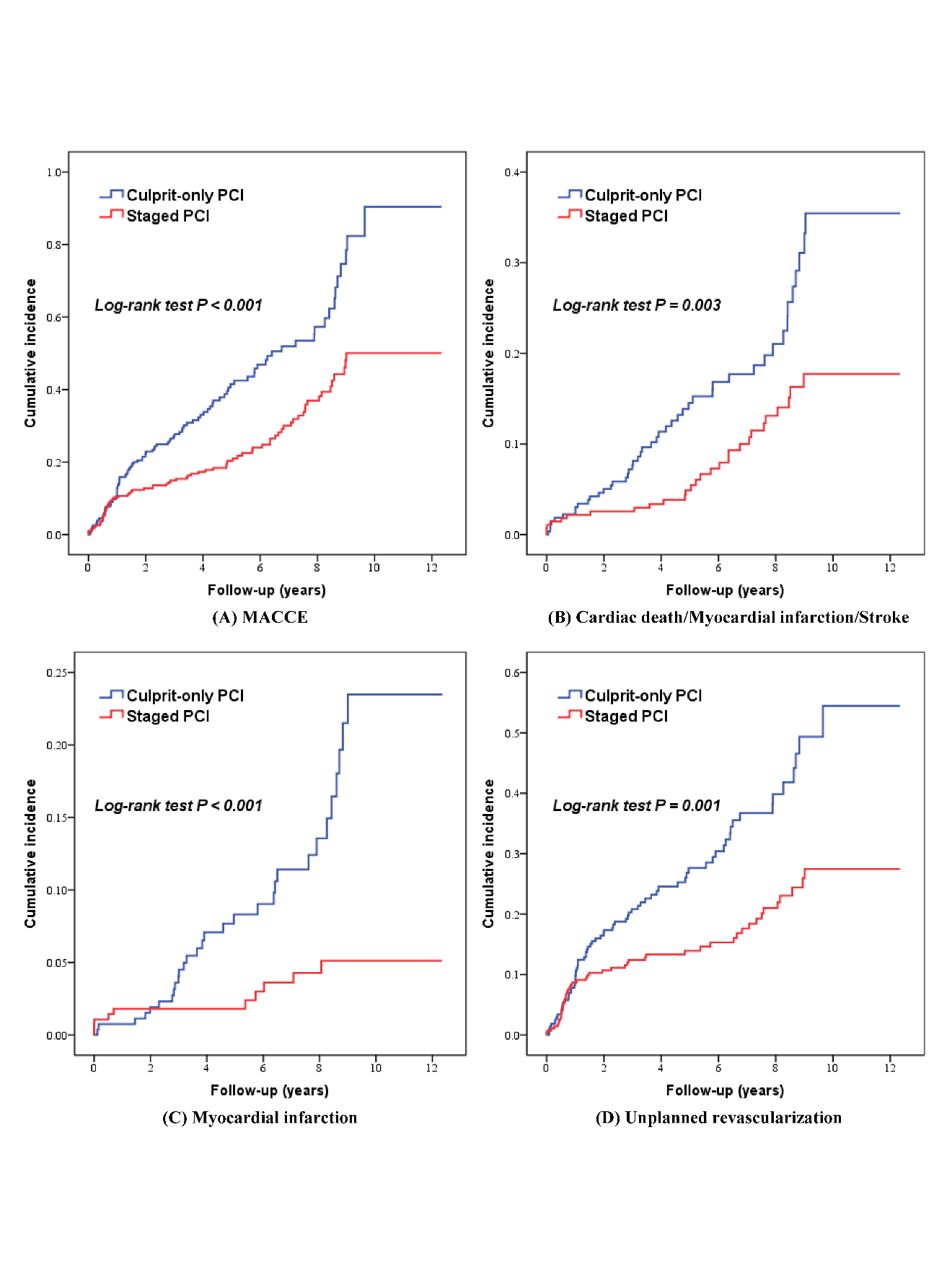
(A)**

**Figure S2** Kaplan-Meier curves of clinical outcomes for **(A)** nondiabetic patients and **(B)** diabetic patients in propensity-matched population.

*MACCE* major adverse cardiac and cerebrovascular event, *PCI* percutaneous coronary intervention.

**
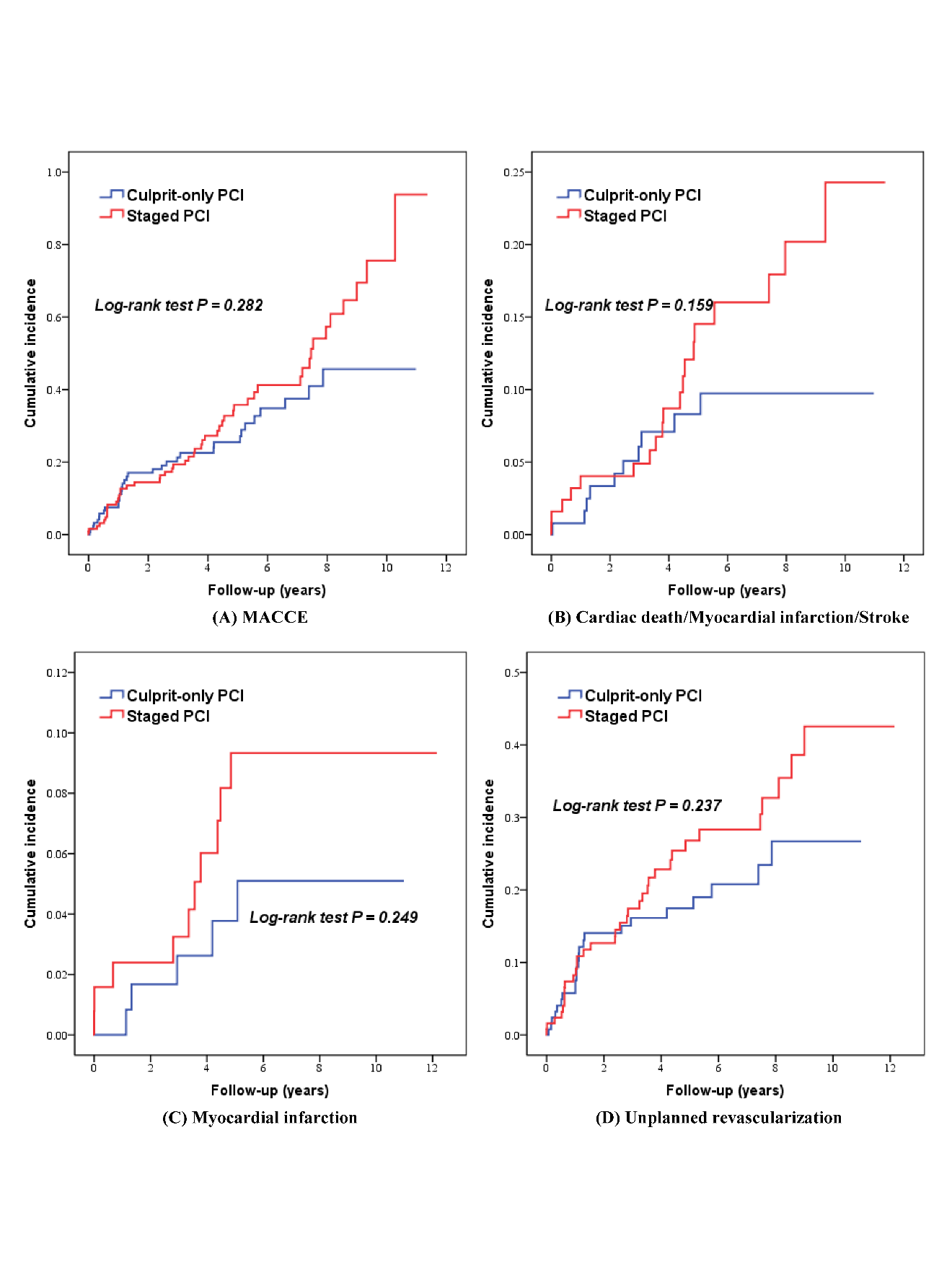
(B)**

**Figure S3** Kaplan-Meier curves of clinical outcomes for **(A)** nondiabetic patients and **(B)** diabetic patients in sensitivity analysis.

*MACCE* major adverse cardiac and cerebrovascular event, *PCI* percutaneous coronary intervention.

**
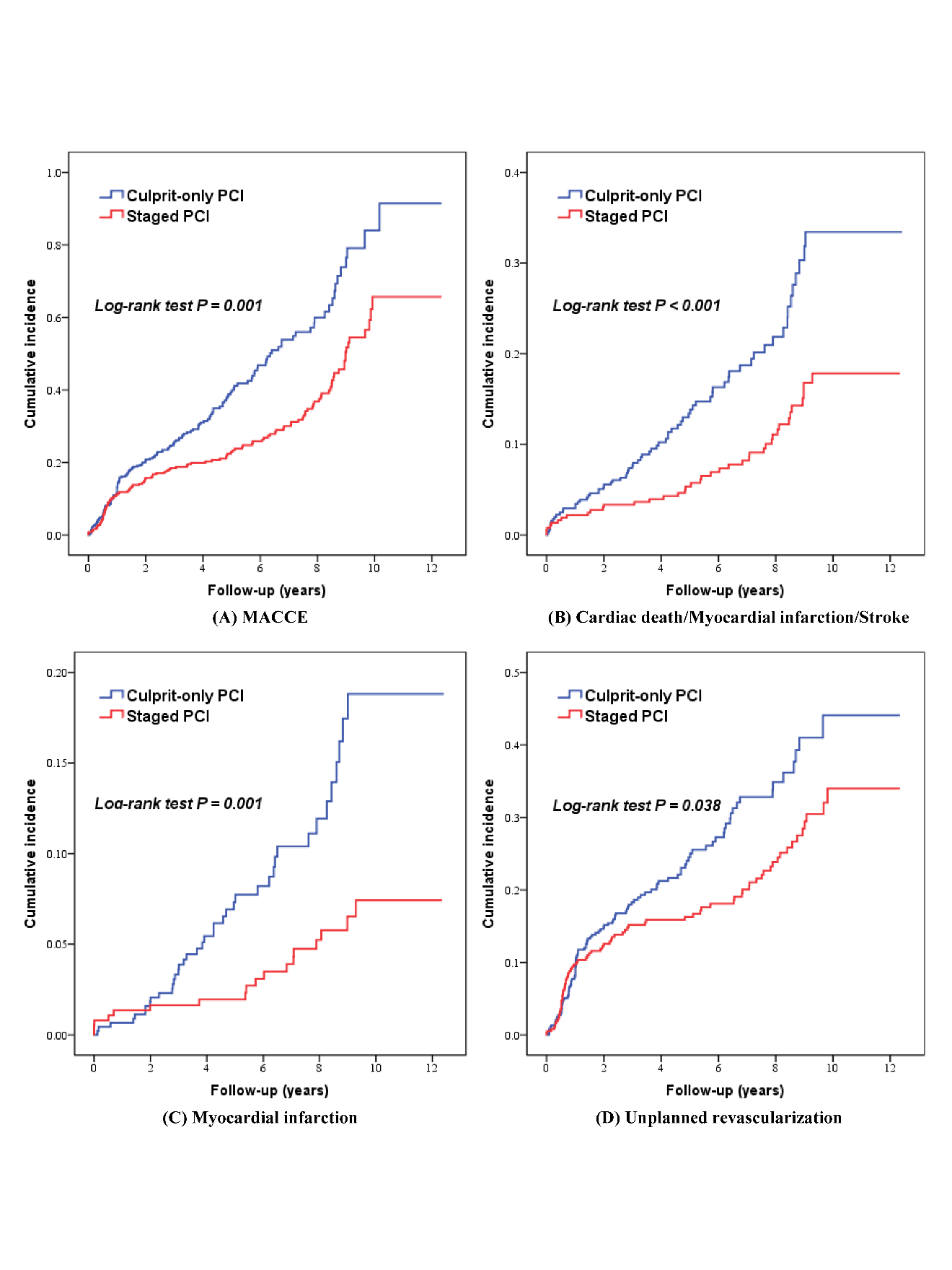
(A)**

**Figure S3** Kaplan-Meier curves of clinical outcomes for **(A)** nondiabetic patients and **(B)** diabetic patients in sensitivity analysis.

*MACCE* major adverse cardiac and cerebrovascular event, *PCI* percutaneous coronary intervention.

**
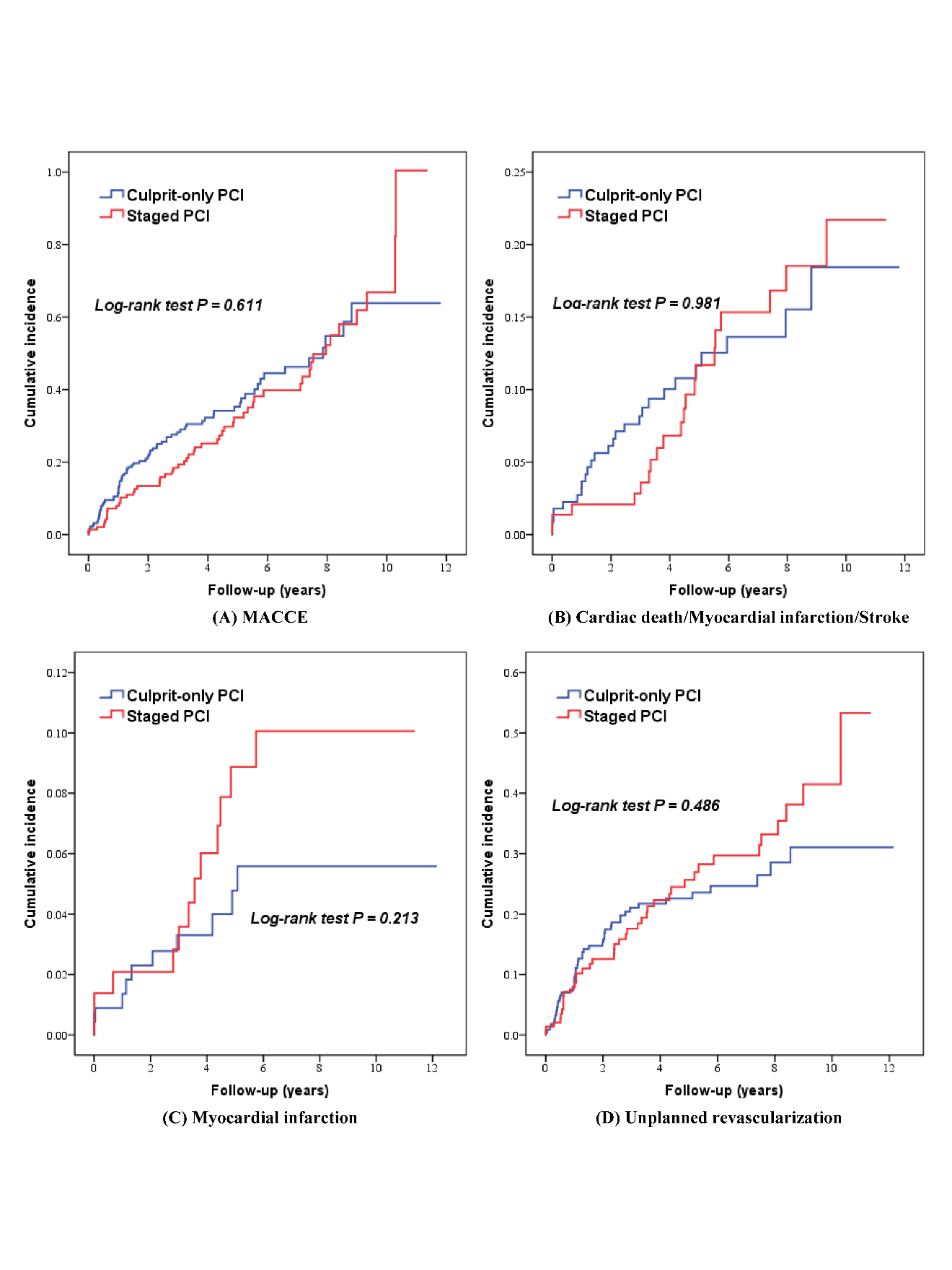
(B)**
